# Statins and the Risk of Gastric Cancer: A Systematic Review and Meta-Analysis

**DOI:** 10.3390/jcm11237180

**Published:** 2022-12-02

**Authors:** Chun-Hsien Su, Md. Mohaimenul Islam, Guhua Jia, Chieh-Chen Wu

**Affiliations:** 1 Department of Exercise and Health Promotion, College of Kinesiology and Health, Chinese Culture University, Taipei 111396, Taiwan; 2 Graduate Institute of Sports Coaching Science, College of Kinesiology and Health, Chinese Culture University, Taipei 111396, Taiwan; 3 International Center for Health Information Technology (ICHIT), Taipei Medical University, Taipei 111396, Taiwan; 4 Sports Teaching Department, Shanghai University of Medicine & Health Sciences, Shanghai 201318, China

**Keywords:** statin, gastric cancer, cancer, diabetes, meta-analysis

## Abstract

Previous epidemiological studies have reported that the use of statins is associated with a decreased risk of gastric cancer, although the beneficial effects of statins on the reduction of gastric cancer remain unclear. Therefore, we conducted a systematic review and meta-analysis to investigate the association between the use of statins and the risk of gastric cancer. Electronic databases such as PubMed, EMBASE, Scopus, and Web of Science were searched between 1 January 2000 and 31 August 2022. Two authors used predefined selection criteria to independently screen all titles, abstracts, and potential full texts. Observational studies (cohort and case-control) or randomized control trials that assessed the association between statins and gastric cancer were included in the primary and secondary analyses. The pooled effect sizes were calculated using the random-effects model. The Meta-analysis of Observational Studies in Epidemiology (MOOSE) reporting guidelines were followed to conduct this study. The total sample size across the 20 included studies was 11,870,553. The use of statins was associated with a reduced risk of gastric cancer (RR_adjusted_: 0.72; 95%CI: 0.64–0.81, *p* < 0.001). However, the effect size of statin use on the risk of gastric cancer was lower in Asian studies compared to Western studies (RR_Asian_: 0.62; 95%CI: 0.53–0.73 vs. RR_western_: 0.88; 95%CI: 0.79–0.99). These findings suggest that the use of statins is associated with a reduced risk of gastric cancer. This reverse association was even stronger among Asian people than Western individuals.

## 1. Introduction

Gastric cancer is a common public health problem associated with a substantial healthcare burden [1]. Gastric cancer ranks fourth in terms of incidence, and is the fifth leading cause of cancer mortality worldwide, with an estimated approximately 1.1 million new cases and 770,000 deaths in 2020 [2,3]. Since a considerable proportion of gastric cancer patients are diagnosed at advanced stages, the five-year survival rate is only 32% [4]. Even though the prevalence and mortality of gastric cancer have been declining in some parts of the world, meaningful prevention strategies are needed to treat it early and reduce the overall healthcare burden. Previous studies have reported that several modifiable (e.g., tobacco smoking, alcohol consumption, obesity, gastroesophageal reflux, and *Helicobacter pylori* (*H. pylori*) infection) and non-modifiable (e.g., age, gender, and ethnicity) risk factors are associated with gastric cancer [5,6,7,8].

Statins are the most commonly prescribed medications and are considered to be effective for lowering cholesterol and protecting against cardiovascular diseases [9,10]. Previous epidemiological studies have highlighted the beneficial effects of statins against cancer [11,12,13,14]. The impact of statin therapy on the risk of gastric cancer, and the relevant biological relationships, which are not entirely understood, have also gained significant attention [15]. Previous studies have reported that statins profoundly reduce tumor growth and mitigation by blocking HMG-CoA reductase [16]. Statins also suppress the expression of genes directly linked to gastric cancer cells and increase apoptosis in early gastric cancer cells [17].

Although statins showed the potential benefits of reducing the risk of gastric cancer, no updated systematic review and meta-analysis of their association has been conducted. This study, therefore, conducted an updated systematic review and investigated the relationship between statin use and the risk of gastric cancer. The evidence of this study will provide adequate answers to this topic and may assist clinicians in weighing the benefits of statins in gastric cancer.

## 2. Methods

This study was based on two main questions. Question 1 was defined as follows: Are patients treated with statins associated with a reduced risk of gastric cancer? Question 2 was defined as follows: Do subgroup analyses support the effect size of the association between statin use and gastric cancer risk? The Preferred Reporting Items for Systematic Reviews and Meta-Analyses (PRISMA) Statement and the guidelines for Meta-analysis Of Observational Studies in Epidemiology (MOOSE) were properly followed to conduct this study [18,19] (Supplementary Table S1).

### 2.1. Databases and Search Strategy

We conducted a comprehensive search on the most popular databases for scientific literatures such as PubMed, EMBASE, Scopus, and Web of Science to obtain all English-language studies published between 1 January 2000 and 31 August 2022. We considered studies that evaluated the association between statin use and the risk of gastric cancer. The following search terms were used: “statin/s” or “hydroxymethylglutaryl-CoA reductase inhibitors” or “lipid-lowering drugs” or “atorvastatin” or “fluvastatin” or “lovastatin” or “pitavastatin” or “pravastatin” or “rosuvastatin” or “simvastatin” and “gastric cancer” or “gastric carcinoma” or “gastric neoplasm” or “stomach cancer” or “stomach carcinoma” or “stomach adenocarcinoma” or “stomach neoplasm” or “cancer” (Supplementary Table S2). Additional searches were conducted in the bibliographies of relevant articles to obtain missing articles.

### 2.2. Inclusion and Exclusion Criteria

All observational studies (e.g., cohort or case-control studies) and randomized control trials (RCTs) that met the following criteria were included: (1) studies that carried out at least one analysis assessing the effect of statins use on gastric cancer, (2) patients with an established diagnosis of gastric cancer by accepted clinical and/or histologic criteria, (3) studies published in English, and (4) studies that provided sufficient information to calculate the pooled effect size. We excluded studies if they were reviews, letters, case reports, or editorials.

### 2.3. Data Extraction

Two authors (CCW and MMI) independently extracted information from all selected studies using piloted data extraction sheets. Extracted data included (1) demographics: author name, publication year, country, (2) population: age, gender, percentage of male, number of statin users, number of gastric cancer patients, (3) methods: study design, inclusion and exclusion criteria, and (4) results: effect sizes (hazard ratio, odds ratio). Any disagreement during the study screening process was resolved by discussing with the third author.

### 2.4. Study Quality Assessment

The risk of bias of RCTs was assessed using the Cochrane Collaboration tool [20], which is comprised of the following domains: (a) sequence generation, (b) allocation concealment, (c) blinding of participants, (d) incomplete outcome data, (e) selective reporting, and (f) other risk of bias. Moreover, the Newcastle-Ottawa Scale was used to assess the methodological quality of the observational studies [21]. The NOS scale is recommended by the Cochrane Handbook for Systematic Reviews of Interventions, and it is divided into three categories such as (a) study selection, (b) comparability, and (c) the ascertainment of exposure (for case-control studies) or the outcome of interest (for cohort studies).

### 2.5. Statistical Analysis

We used ORs and HRs to calculate the overall pooled effect sizes of statin use on the risk of gastric cancer. The pooled RRs were calculated using random-effects models. We calculated heterogeneity across studies using the Q-statistic and quantified using the inconsistency I^2^. The I^2^ values were classified into four groups: of 0~29%, 30~49%, 50~74%, and 75~100%, representing very low, low, medium, and high inconsistency, respectively [22,23,24]. We also conducted subgroup analyses to assess the potential impact of study design, region, quality of observational studies (NOS ≤ 7 vs. NOS > 7), duration, and statin types. In the sensitivity analysis, the impact of each study on the summary statistics was evaluated by excluding one study at a time from the meta-analysis. A forest plot was drawn to visually represent the effect size of each study and the pooled analyses. Finally, funnel plots and the Egger regression test of funnel plot asymmetry were used to calculate overall publication bias. The statistical analyses were performed using comprehensive meta-analysis software (CMA). A *p* < 0.05 was considered statistically significant.

## 3. Results

### 3.1. Study Selection and Characteristics

**Figure 1:**
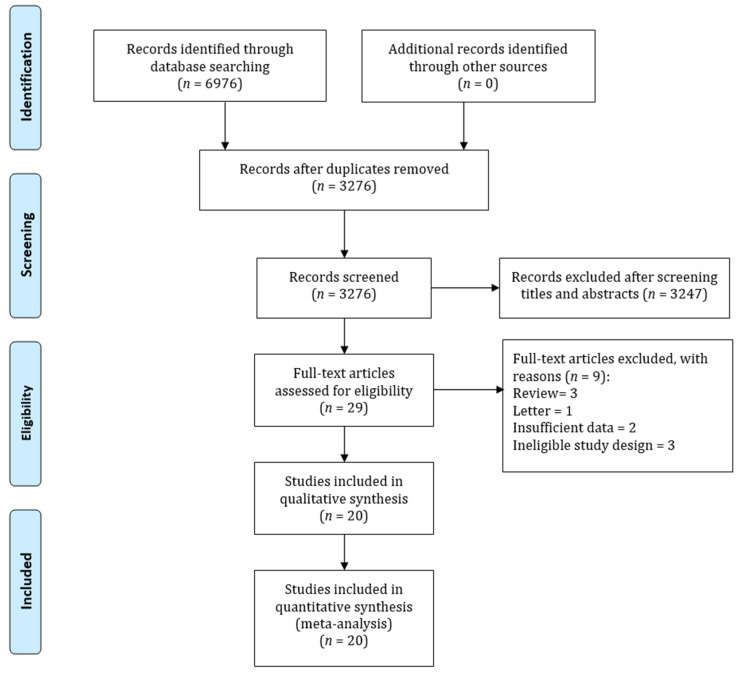
PRISMA flowchart for summarizing study identification and selection.

Figure 1 shows the PRISMA diagram of the selection of studies. The search identified 6976 studies. However, 3700 studies were excluded for duplications and 3247 studies were excluded after screening the titles and abstracts. In total, 29 studies were selected for full-text screening based on prespecified selection criteria. Finally, 20 studies were selected for the meta-analysis [17,25,26,27,28,29,30,31,32,33,34,35,36,37,38,39,40,41,42,43].

These 20 studies consisted of 9 cohort, 8 case-control, and 3 RCTs studies, which comprised of 11,870,553 participants. Twelve studies were conducted in Asia and eight studies were conducted in Western countries. A total of 17 out of 20 studies used administrative databases to identify statin users and gastric cancer patients. More than half of observational studies were of good quality, reflected by a Newcastle-Ottawa score of at least 8. Table 1 presents the characteristics of the included studies, intervention, outcomes, included and excluded criteria, and study quality.

### 3.2. Statin Use and the Risk of Gastric Cancer

**Figure 2:**
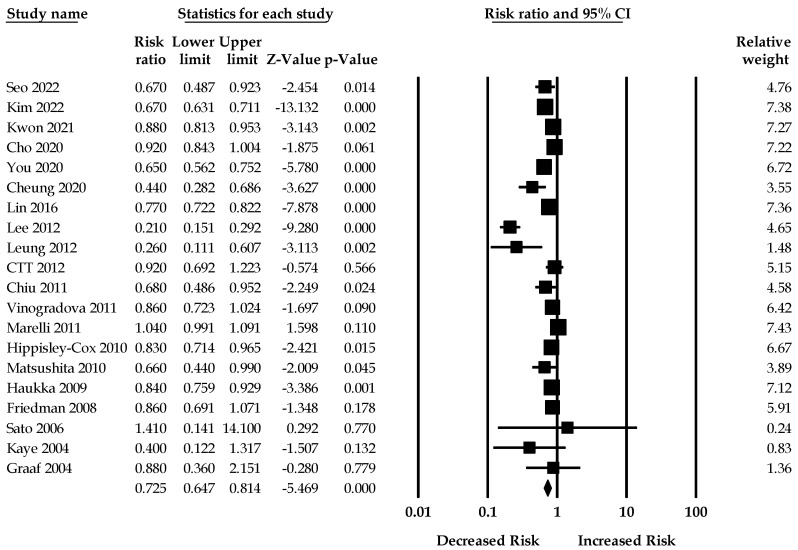
Association between statin use and the risk of gastric cancer [17,25,26,27,28,29,30,31,32,33,34,35,36,37,38,39,40,41,42,43].

Twenty studies (three RCTs and 17 observational studies) reporting the effect of statins on gastric cancer were included in the meta-analysis. When all adjusted effect sizes were pooled, the summary RR in statin users compared with statin nonusers from the random-effects models was 0.72 (95%CI: 0.64–0.81). There was significant heterogeneity across studies (I^2^ = 92.63, Q = 272.69, τ^2^ = 0.05) in the random-effects model. Figure 2 shows the forest plot of the association between statin use and the risk of gastric cancer.

### 3.3. Sensitivity and Subgroup Analyses

We also conducted a sensitivity analysis to assess changing the overall effect sizes of the use of statin on gastric cancer and the presence of heterogeneity by omitting one study from the main analysis (Supplementary Table S3). The overall effect size and heterogeneity did not change after sensitivity analyses.

The pooled RRs from the random-effects model based on data from cohort studies, case-control studies, and RCTs were 0.77 (95%CI: 0.66–0.90), 0.61 (95%CI: 0.48–0.77), and 0.82 (95%CI: 0.65–1.04), respectively. However, there was significant heterogeneity across the studies (Table 2).

The pooled RRs for the studies from Asia and Western countries were 0.62 (95%CI: 0.53–0.73; I^2^ = 92.67, Q = 150.22, τ^2^ = 0.05) and 0.88 (95%CI: 0.79–0.99, I^2^ = 72.27, Q = 25.25, τ^2^ = 0.01), respectively.

The pooled RRs for male and female patients were 0.80 (95%CI: 0.68–0.94), and 0.72 (95%CI: 0.55–0.94), respectively. However, there was moderate heterogeneity across the studies.

### 3.4. Dose–Response Association between Statins and Gastric Cancer

Kim et al. [26] evaluated the risk of gastric acid among statin users who received <1460 and ≥1460 cumulative defined daily doses (cDDDs). After propensity score matching, the effect size for the association between gastric cancer risk and statin users with <1460 cDDDs and ≥1460 cDDDs were 0.98 (95%CI: 0.91–1.06) and 0.22 (95%CI: 0.19–0.26), respectively. Cho et al. [27] classified the cumulative duration of statin use into four categories such as <182.5, 182.5–365.0, 365.0–547.5, and 547.5–730.0. The risk of gastric cancer decreased as cDDDs increased among statin users [HR0.96 (95%CI: 0.89–1.04), 0.85 (95%CI: 0.74–0.96), 0.78 (95%CI: 0.68–0.90), and 0.89 (95%CI: 0.82–0.96)]. Finally, Chiu et al. [34] examined the risk of gastric cancer among various statin users, and they observed a significant trend of reducing gastric cancer with increasing cumulative dose. The adjusted ORs were 0.90 (95% CI = 0.60–1.36) for the group with cumulative statin use <134.25 DDDs and 0.49 (95% CI = 0.30–0.79) for the group with cumulative statin use of ≥134.25 DDDs.

### 3.5. Duration–Response Association between Statins and Gastric Cancer

Cheung et al. [29] invested the duration–response relationship and categorized statin users into two groups: (i) <5 years and (ii) ≥5 years. Lower risk of gastric cancer was observed among patients who used statins longer (0.46 [95%CI:0.25–0.86] for <5 years of use and 0.43 [95%CI:0.29–0.66] for ≥5 years of use). Le et al. [31] also evaluated the impact of duration of statin use on gastric cancer risk. Diabetes patients who received statins less than 1 year before the index date had an effect size of 0.45; however, the risk of gastric cancer was even lower among patients with statin use of more than 2 years (0.154 [95%CI: 0.09–0.26]).

### 3.6. Publication Bias

**Figure 3:**
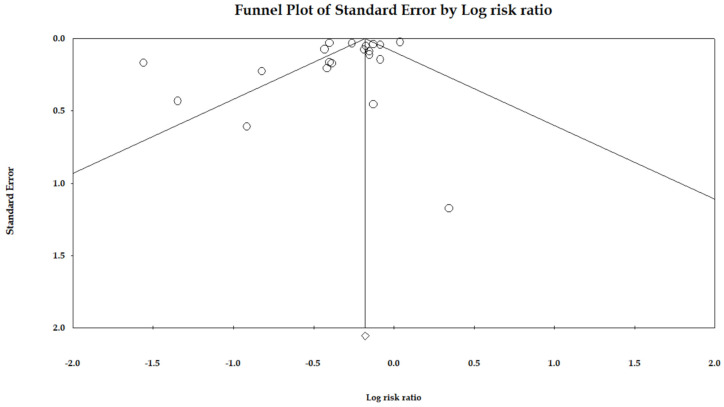
Funnel plot of the association between statin use and risk of gastric cancer.

Figure 3 shows the funnel plot for publication bias. Out of 20 data points, six lay outside the triangle, and only one lay on the left side of the triangle altitude. The Egger regression test of the funnel asymmetry showed no publication bias (*p* = 0.09).

## 4. Discussion

In this updated systematic review and meta-analysis, we evaluated the association between statin use and the risk of gastric cancer, which is currently unclear. Our findings are similar to previous evidence [15,44,45,46,47] that showed statin use is associated with a significantly reduced risk of gastric cancer. The strength of this association varied among study designs, and no association was observed in the RCTs. In addition, a more protective effect was observed in Asian people compared to Western individuals.

The possible biological mechanisms as to how statin use reduces the risk of gastric cancer is still unclear. However, several biological pathways may help to understand the process of their association. Statin reduces the production of cholesterol, dolichol, and coenzyme Q10 by inhibiting HMG-CoA reductase in the mevalonate pathway [48,49]. Previous studies highlighted that statin increases apoptosis, suppress angiogenesis, and changes the tumor microenvironment downstream of the mevalonate pathway [50,51,52]. Moreover, suppression of the mevalonate pathway due to statin use can reduce radiosensitization or chemosensitization [53,54]. Statins interrupt the production of primary geranylgeranyl pyrophosphate (GGPP) and farnesylpyrophophosphate (FPP) and delay the growth of malignant cells, eventually leading to apoptosis [55]. Statins alter the activation of the proteasome pathway, inhibiting the breakdown of both p21 and p27 [56]. Finally, statins allow p21 and p27 molecules to utilize their growth-inhibitory effects and try to slow down gastric cancer cell mitosis [57].

The effect size of the relationship between statin use and the risk of gastric cancer was different in Asian and Western population. Statin use significantly showed a reduction of gastric cancer in Asian populations, but no association was observed in Western populations. Previous studies assessed statin responses between Asians and Westerners (European Americans), showing a difference in pharmacokinetic and pharmacodynamic effects [58,59]. Studies highlighted that statin responses were significantly different even after adjustment for potential confounding factors such as age, comorbidities, and/or socioeconomic status. Body size differences between Asians and Westerners may also contribute to pharmacokinetic variation (<10%) [60,61]. Moreover, genetic variation among Asians can influence statin pharmacokinetics and pharmacodynamics [62].

The findings of our study also showed that the reduction of gastric cancer among statin users was higher in the case-control studies compared to cohort and RCTs. The inherent risk of bias among case-control studies is consistently high due to potential confounding factors. In the cohort studies, maximum efforts are given to reduce the possible biases, although it does demonstrate a causal relationship. In the clinical-decision making, RCTs are considered the gold standard of study design, revealing the causal relationship between a drug and gastric cancer risk. On the other hand, more chemo-preventive effects of statins were observed in the low-quality studies, which may overestimate their true effect. Low-quality studies often contain a lack of random patient allocation and a lack of adjusting for numerous covariates; therefore, it is not possible to eliminate the possibility of residual confounding factors. Included studies also demonstrated that statins are even more protective against gastric cancer if patients take a higher dose over a longer duration of time [26,27,28]. Kwon et al. [17] showed that a significant reduction in gastric cancer was observed among patients taking hydrophilic statins. Our subgroup analyses showed that pravastatin (hydrophilic) was associated with an insignificant reduction of gastric cancer, whereas rosuvastatin showed an increased risk of gastric cancer. More studies with controlled confounding factors and larger follow-up periods are needed to provide supporting evidence that demonstrates the effectiveness of statins against gastric cancer.

There are several strengths in this study. First, this is the most updated systematic review and meta-analysis so far that evaluated the relationship between statin use and gastric cancer risk. We included both RCTs and observational studies to summarize overall effect size that ensures the best available evidence through a meta-analytic approach. Second, this meta-analysis included a large sample size and the quality of included studies were high. Therefore, the findings of this study were more reliable. Third, we could identify statin as an independent risk factor for gastric cancer since we adjusted with potential confounding factors to estimate the pooled summary size.

This study also has several limitations. First, most studies were observational studies with a highly heterogeneous population with different patient characteristics. The heterogeneity among the studies was high, although this can be explained by study design, region, and study quality. Second, there was limited information regarding the types of statins, indication, follow-up, and duration of statins; therefore, we were unable to pool effect size based on dosage, duration, and follow-up. Third, several potential covariates/confounders related to gastric cancer risk have not been adjusted in the included study. Future studies should adjust all possible confounders to pool the effect sizes and to show a stronger association between statin use and gastric cancer risk.

## 5. Conclusions

The findings of this updated meta-analysis suggest that statin use is associated with a reduced risk of gastric cancer. Although the pooled effect size of observational studies showed a possible role of statin therapy in the prevention of gastric cancer, the pooled effect size of the clinical trials and the risk of the bias of observational studies do not encourage physicians to consider statin use to achieve a reduction in gastric cancer. More RCTs with larger populations, longer follow-up, and standardized methods are required to consider statin use as a strategy for reducing gastric cancer.

## Figures and Tables

**Table 1:** Basic characteristics of included studies.

Author [Reference]	Year	Country	Study Design	Study Participant	Age (Year)	Gender (Female)	Inclusion Criteria for Gastric Cancer	Adjustment	NOS/ ROB
Seo [25]	2022	South Korea	Cohort	1,025,340	Range	51.4/51.2	ICD	Age, sex, smoking, DM, GERD, hypertension, hyperlipidemia, HF	8
Kim [26]	2022	South Korea	Cohort	1,008,101	Range	58.2/58.5	ICD	Age, sex, CCI, CHF, DM, Stroke, hypertension, aspirin use	8
Kwon [17]	2021	South Korea	C-C	8798	Range	26.82/26.83	ICD	Age, sex, CCI, BMI, smoking, alcohol, CCI	8
Cho [27]	2020	South Korea	Cohort	1,740,975	Range	41.44	ICD	Age, sex, CCI, BMI, smoking, alcohol, hypertension	8
You [28]	2020	South Korea	Cohort	31,149	M = 52.6 F = 58.7	46.74	ICD	Age, smoking, BMI, total cholesterol, DM, hypertension	8
Cheung [29]	2020	Hong Kong	Cohort	63,605	61.7/63.6	51.1/52.5	ICD	Age, sex, gastric ulcer, DM, IHD, CHF, CRF	8
Lin [30]	2016	Taiwan	C-C	39,455	Range	37/37	ICD	Age, sex, H. pylori, gastric disease, GERD, gastritis	8
Lee [31]	2016	South Korea	C-C	2949	62.6/62.6	23.5/23.5	ICD	Age, sex, other medications	5
Leung [32]	2012	Taiwan	C-C	34,205	Range	53.69/52.56	ICD	Age, sex, CCI, DM, others lipid lowering agents	7
CTT [33]	2012	Europe/USA/ Australia	RCT	134,729	NR	NR	Adverse event reporting	Variable	Low
Chiu [34]	2011	Taiwan	C-C	1665	69.64/69.51	NR	ICD	Age, sex, Helicobacter pylori eradication, gastric ulcer, peptic ulcer, duodenal ulcer	8
Vinogradova [35]	2011	UK	C-C	450,379	Range	47.4/47.0	ICD	BMI, smoking, MI, CHF, DM, hypertension, stroke	8
Marelli [36]	2011	USA	Cohort	91,000	Range	47.77/47.43	ICD	Age, sex, race, BMI, smoking	7
Hippisley-Cox [37]	2010	UK	Cohort	2,004,692	57.2/44.4	46.4/51.1	ICD	Age, sex, race, BMI, smoking, AF, CCF, DM, hypertension	7
Matsushita [38]	2010	Japan	RCT	13,819	58	48	Adverse event reporting	Variable	Low
Haukka [39]	2009	Finland	Cohort	946,629	Range	50.08	ICD	Age, sex	7
Friedman [40]	2008	USA	Cohort	4,222,797	Range	NR	ICD	Other medications (aspirin/NSAIDs)	6
Sato [41]	2006	Japan	RCT	267	NR	18.25	NR	Age, sex, smoking	Low
Kaye [42]	2004	UK	C-C	18,127	Range	48.9/49.8	ICD	Age, sex, BMI, smoking, region	8
Graaf [43]	2004	Netherland	C-C	20,209	Range	51/51	ICD	Age, sex, other medications, others lipid lowering agent, region	7

Note: C-C: Case-control; RCT: Randomized control trial; ICD: International Classification of Diseases; DM: Diabetes mellitus; GERD: Gastroesophageal reflux disease; MI: Myocardial infarction; BMI: Basal metabolic index; CHF: Congestive heart failure; AF: Atrial fibrillation; CCF: Congestive cardiac failure; IHD: Ischemic heart disease; CCI: Charlson Comorbidity Index; NR: Not reported; NSAIDS: Non-steroidal anti-inflammatory drugs; NOS: Newcastle-Ottawa Scale; UK: United Kingdom; USA: United States of America; M: Male; F: Female.

**Table 2:** Subgroup analyses for the association between statin use and risk of gastric cancer.

Study	No of Studies	Pooled Estimates		Test of Heterogeneity		
		RR (95%CI)	*p*-Value	Q Value	*p*-Value	I^2^ (%)
All Studies	20	0.72 (0.64–0.81)	<0.001	245.26	<0.001	92.25
Study Design						
Cohort	9	0.77 (0.66–0.90)	0.001	155.32	<0.001	94.84
Case-control	8	0.61 (0.48–0.77)	<0.001	79.25	<0.001	91.16
RCT	3	0.82 (0.65–1.04)	0.11	1.93	0.38	0
Region						
Asian	12	0.62 (0.53–0.73)	<0.001	150.22	<0.001	92.67
Western	8	0.88 (0.79–0.99)	0.02	25.25	0.001	72.27
Gender						
Male	3	0.80 (0.68–0.94)	0.008	15.14	<0.001	53.77
Female	3	0.72 (0.55–0.94)	0.017	20.69	0.004	66.17
Methodological Quality						
Low	7	0.64 (0.48–0.87)	0.004	144.20	<0.001	95.83
High	10	0.74 (0.66–0.82)	<0.001	65.32	<0.001	84.69
Adjusted with Age						
Yes	16	0.68 (0.60–0.78)	<0.001	270.57	<0.001	94.08
No	4	0.85 (0.75–0.95)	0.007	1.82	0.61	0
Adjusted with BMI						
Yes	7	0.85 (0.75–0.95)	0.007	51.31	<0.001	86.35
No	13	0.63 (0.54–0.74)	<0.001	109.68	<0.001	89.06
Adjusted with Smoking						
Yes	9	0.83 (0.74–0.93)	0.002	55.75	<0.001	83.85
No	11	0.63 (0.53–0.74)	<0.001	109.18	<0.001	90.84
Adjusted with Diabetes						
Yes	7	0.69 (0.60–0.78)	<0.001	23.11	0.002	69.71
No	13	0.75 (0.65–0.78)	<0.001	169.83	<0.001	92.93
Adjusted with Hypertension						
Yes	5	0.79 (0.66–0.93)	0.007	42.28	<0.001	90.54
No	16	0.68 (0.58–0.79)	<0.001	204.55	<0.001	92.66
Type of statin						
Simvastatin	2	0.76 (0.71–0.83)	<0.001	0.50	<0.001	0
Lovastatin	1	0.79 (0.72–0.86)	<0.001	-	-	-
Atorvastatin	1	0.80 (0.60–1.06)	0.12	-	-	-
Pravastatin	1	0.71 (0.36–1.37)	0.31	-	-	-
Fluvastatin	1	1.02 (0.45–2.29)	0.96	-	-	-
Rosuvastatin	1	1.35 (0.64–2.85)	0.42	-	-	-

## Data Availability

Not applicable.

## Supplementary Materials

The following supporting information can be downloaded at: https://www.mdpi.com/article/10.3390/jcm11237180/s1, Table S1: PRISMA checklist; Table S2: Search strategy; Table S3: Sensitivity analyses for the association between statin use and the risk of gastric cancer. Reference [63] has been cited in Supplementary Materials.
